# Modulating Ion Behavior by Functional Nanodiamond Modified Separator for High-Rate Durable Aqueous Zinc-Ion Battery

**DOI:** 10.1021/acsami.4c15737

**Published:** 2024-12-09

**Authors:** Qiuxia Zhang, Linfeng Wan, Xuan Gao, Shaoheng Cheng, Nan Gao, Claire J. Carmalt, Yuhang Dai, Guanjie He, Hongdong Li

**Affiliations:** † State Key Laboratory of Superhard Materials, College of Physics, Jilin University, Changchun 130012, PR China; ‡ Christopher Ingold Laboratory, Department of Chemistry, University College London, London, WC1H 0AJ, U.K.; § Thom Building, Department of Engineering Science, University of Oxford, Oxford, OX1 3PJ, U.K.

**Keywords:** Aqueous zinc-ion batteries, Separator modifications, Electrode−electrolyte interface, Nanodiamond, Zn^2+^ transport properties

## Abstract

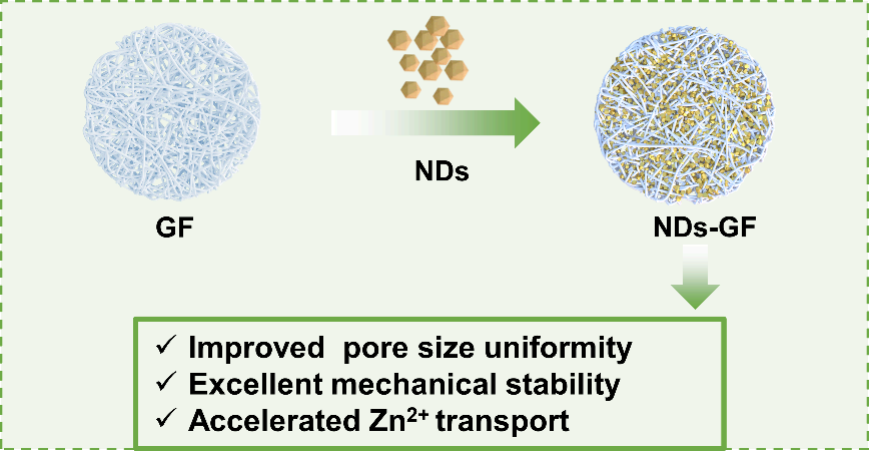

Aqueous zinc-ion batteries (AZIBs) have garnered widespread attention due to their promising development and application prospects. However, progress of AZIBs has been hindered by zinc (Zn) dendrites and side reactions at the electrode–electrolyte interface (EEI). In particular, the large and uneven pores of commercial glass fiber (GF) separators lead to nonuniform Zn^2+^ transport, which causes side reactions. In this study, we employed nanodiamonds (NDs) to regulate the separator pore structure and utilized its surface oxygen-containing functional groups to control the Zn^2+^ transport properties. Due to their excellent chemical inertness, superhardness, ultrahigh thermal conductivity, and abundant surface functional groups, NDs modified GF separators for dendrite-free and high-performance AZIBs. Experimental outcomes demonstrate that Zn||Zn symmetric cells using NDs-GF separators exhibit regular charge–discharge profiles, minimal fluctuations, and an ultralong cycling lifespan of nearly 1800 h under a current density of 5 mA cm^–2^ with a capacity density of 1 mAh cm^–2^ and 240 h under a high current density of 10 mA cm^–2^ with a capacity density of 10 mAh cm^–2^. The Zn||MnO_2_ full cells using NDs-GF separators showcase a high retention after 1000 cycles at 1 A g^–1^. This research proposes a modification method for developing advanced separators in AZIBs technology.

## 1 Introduction

Metal-based energy storage systems (ESS) have garnered heightened research attention owing to their remarkable electrochemical performance characteristics.^1^ In contradistinction to lithium (Li), sodium (Na), and potassium (K), zinc (Zn) metal exhibits distinct advantages in terms of significant theoretical capacity (820 mAh g^–1^) and cost-effectiveness.^2^ Zn metal has a notably valuable anode in aqueous zinc-ion batteries (AZIBs) due to the relatively low potential (−0.76 V vs the standard hydrogen electrode (SHE)), which enables stable operation within aqueous electrolytes.^3,4^ During the charging–discharging process, Zn^2+^ shuttles through the pores of the separator filled with electrolyte and continuously deposits and dissolves on the Zn anode surface. However, due to the influence of various factors such as electrodes, electrolytes, and separators, Zn^2+^ is prone to form Zn dendrites, a passivation layer, and even corrosion during the Zn stripping/plating process, which urgently needs to be addressed.^5,6^

At present, research mainly focuses on the improvement of cathode materials, protection of Zn anodes, optimization of electrolytes, etc. However, there is still relatively little research on separators. Inside the cell structure, although the separator does not take part in the electrochemical reaction, it does not mean that the separator is not important in AZIBs.^7−9^ The function of separators is to prevent the electrode contact from causing the cell short circuit, absorb the electrolyte for Zn^2+^ transport, control the Zn^2+^ transport rate by pore size, and regulate the uniform Zn^2+^ deposition and dissolution, thereby reducing Zn dendrites and byproducts.^10^ Generally speaking, the ideal separator should have the characteristics of moderate porosity, low internal resistance, high ion conductivity, and excellent stability.^11^ The GF separator has a low conductivity, large porosity, high ion conductivity, and fine wettability for aqueous electrolytes. Especially, it can absorb 360% electrolyte and achieve high ion conductivity (generally 17.3 mS cm^–1^).^12^ Therefore, the GF separator stands out among various separator materials and becomes the most commonly used choice for studying AZIBs. Nevertheless, the uneven pore structure of commercial GF separators can lead to nonuniform ion transport rates, which can induce the Zn dendrites and byproducts. In addition, GF separators lack excellent mechanical properties, making it difficult to ensure the integrity of the separator under high current density, resulting in the separator being punctured by Zn dendrites, leading to short circuits and cell failure.^10,13^ Similar to electrodes and electrolytes, GF separators also show enormous research potential and can be modified and innovated by different research strategies. There are a few works reported on regulating GF separators by modifying high mechanical strength sulfonated poly arylene ether sulfone (SPAES)^14^ and porous zeolite imidazole salt framework-8 (ZIF-8),^15^ etc., to promote the desolvation of hydrated Zn^2+^, guide uniform Zn^2+^ transfer, facilitate dendrite-free, inhibit production of hydrogen gas, and improve reversible Zn^2+^ plating/stripping process. Although these attempts have helped boost performances (capacity, dendrite-free, reduced byproducts, and long-term stable cycling) of AZIBs, multiple issues of process complexity, side effects, increase in weight, incompatibility with production line, cost-effectiveness, etc., should be fully taken into account and efficiently addressed.

Nanodiamonds (NDs) with unique properties of excellent chemical inertness, superhardness, ultrahigh thermal conductivity, and abundant surface functional groups have been applied in lithium-ion and sodium-ion batteries, to improve the performance of these second-ion batteries.^16,17^ Recently, NDs have been used as a protective material for Zn anode in AZIBs, effectively suppressing side reactions (Zn dendrite, corrosion), and improving the cycling stability of batteries.^18,19^ In this work, we fabricate a NDs-modified GF (NDs-GF) separator for regulating the Zn^2+^ deposition behavior, achieving a high rate and durable AZIBs. There, the role of NDs is mainly reflected in the following aspects: (1) NDs fill and improve the uniformity of GF separator pore size, controlling the uniformity of Zn^2+^ transport, which inhibits the formation of Zn dendrites. (2) Benefiting from the excellent mechanical stability, it can enhance the puncture resistance and maintain good structural stability of the GF separator inside the cell.^20^ (3) Benefiting from the ultrahigh thermal conductivity (∼2000 Wm^–1^ K^–1^), it can timely conduct the heat generated during the redox reaction process, prevent local overheating of the cell, and thus improve thermal safety and stability.^21^ (4) Benefiting from the abundant functional groups such as hydroxyl (−OH) and carboxylic (−COOH), −OH can reduce the H^+^ concentration at EEI by forming hydrogen bonds, inhibiting hydrogen evolution reaction (HER). Meanwhile, the unshared electron pair of −COOH can form electrostatic interactions with Zn^2+^, accelerate the Zn^2+^ transport, assist in the Zn^2+^ desolvation process, and improve reaction kinetics.^22,23^

The GF separators modified by NDs have suitable pore structure and excellent mechanical and chemical properties, which is beneficial for the rapid and uniform Zn^2+^ transport at the electrode–electrolyte interface (EEI). Therefore, the NDs-GF separator significantly increases the Zn^2+^ transference numbers (*t*_Zn^2+^_) (0.45 vs 0.13), accelerating the Zn^2+^ desolvation process. In addition, the Zn||Zn symmetric cells using NDs-GF separators manifest consistent charge–discharge curves without evident fluctuation and establish an extraordinarily prolonged cycle lifespan of nearly 1800 h. The Zn||MnO_2_ full cells using NDs-GF separators exhibit a higher storage capacity.

## 2 Experimental Section

### 2.1 Separator, Electrolyte, and Electrode Preparation

The NDs water-based dispersion was obtained by adding 5 mg of NDs into 30 mL of deionized water and ultrasonication for 1 h. Then, the GF separator was completely immersed in the NDs water-based dispersion, allowed to stand for 10 min, and dried in a drying oven (60 °C for 12 h), which is a GF separator modified with medium-content NDs, as the NDs-GF separator. The GF separator was modified with low-content NDs and high-content NDs, and their experimental steps were the same as the above experimental steps, except that the amount of deionized water used was 10 and 50 mL, respectively.

The ZnSO_4_·7H_2_O (5.751 g) was dissolved in deionized water (10 mL) to form a 2 M ZnSO_4_ electrolyte. In addition, the MnSO_4_·H_2_O (0.338 g) was dissolved in the prepared 2 M ZnSO_4_ electrolyte, as a mixed electrolyte of 2 M ZnSO_4_ and 0.2 M MnSO_4_, which as the electrolyte for the Zn||MnO_2_ full cell.

The preparation of the MnO_2_ cathode was as follows: the mass ratio of MnO_2_, Super P, and PVDF powders was 7:2:1, mixed and ground in a mortar and stirred into a slurry using *N*-methyl-2-pyrrolidine (NMP). The stirred slurry was evenly cast on the current collector (carbon paper) and dried at 80 °C for 12 h in a vacuum drying oven. After it was dried, it was taken out and cut into small circular pieces (Φ = 12 mm), and the mass load of MnO_2_ was 2.1 mg cm^–2^, which is named the MnO_2_ cathode.

### 2.2 Material Characterizations

The surface morphology and elements of GF, NDs-GF separators, and Zn anode surfaces were characterized by scanning electron microscopy (SEM, S-4800, Hitachi Limited) and energy dispersive spectroscopy (EDS). The discharge products of the MnO_2_ cathode after cycling were characterized by X-ray diffraction (XRD) spectroscopy. The NDs and NDs-GF separators were characterized by transmission electron microscopy (TEM, JEM-2100F, JEOL). The spectra of NDs were recorded by a Fourier transform infrared (FTIR, Nicolet-670) spectrometer. The pore size distribution of separators was measured by the Brunauer–Emmett–Teller method (JW-BK200). The mechanical properties of the NDs-GF and GF separator were tested via AGXplus at a stretching speed of 1 mm min^–1^. The wettability of the GF and NDs-GF separators corresponding to the electrolyte was tested with the XG-CAMC33 system (SUNZERN, China).

### 2.3 Cells Preparation and Electrochemical Measurements

The assembled Zn||MnO_2_ full cell using Zn and MnO_2_ cathode, the assembled Zn||Cu or Zn||Ti asymmetric cells using Zn and Cu or Ti, and the assembled Zn||Zn symmetric cells using Zn and Zn, with an electrolyte amount of 150 μL. Evaluate the electrochemical performance of GF and NDs-GF separators by CR2025 coin cells. The electrochemical workstation (Shanghai Chenhua CHI660E) conducted the following tests: Electrochemical impedance spectroscopy (EIS) test under a frequency range of 0.01 Hz–100 kHz; Cyclic voltammetry (CV) test under a scanning rate of 0.1–1 mV s^–1^ and the voltage range of 0.8–1.9 V; Linear Sweep Voltammetry (LSV) test; Tafel plots test; Chronoamperometry (CA) test under −150 mV; amperometric *I–t* test. The galvanostatic charge–discharge cycle test under the voltage range of 0.8–1.9 V by a land battery measurement system (CT2001 1 A).

## 3 Results and Discussion

**Figure 1:**
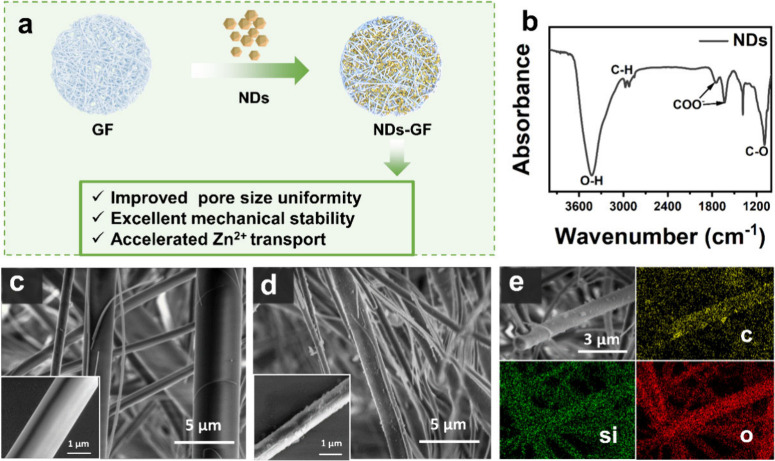
(a) Schematic illustration of the NDs modified GF separator. (b) FTIR spectra of the NDs. (c) SEM of the GF. (d, e) SEM and the corresponding EDS of the NDs-GF separator.

For the preparation of NDs-GF separator (Figure 1a), a commercial GF separator was immersed in an aqueous solution of NDs (commercial detonation NDs with size of 5–10 nm (Figure S1))^24^ and then dried in a drying oven at 60 °C. Subsequently, we conducted some simple characterization tests and relevant analysis on the NDs-GF separator. The ND surface is functionalized by −OH and −COOH groups, determined using Fourier Transform infrared (FTIR) spectroscopy of NDs (Figure 1b). The characteristic peaks of the FTIR appear at approximately 3433 cm^–1^, 1630/1758 cm^–1^, and 1089 cm^–1^, which correspond to the stretching vibrations of the H–O bond, the COO- bond, and the C–O bond, in turn.^19^ As shown in Figure 1c, the scanning electron microscopy (SEM) image of the GF separator is composed of longer glass fibers with great length-diameter ratio and smooth surfaces, and importantly, both the shapes and sizes of the pores are nonuniform. For the NDs-GF separator (Figure 1d), a large amount of aggregated NDs are attached to the fibers, enlarging the specific surface area and reducing the pore size of the separator, which is conducive to “trapping” more electrolytes and accelerating the Zn^2+^ rapid transport.^25^ The test results indicate that the average pore size for N_2_ adsorption of the NDs-GF separator surface was 5.91 nm (Figure S2), which is slightly smaller than that of the GF separator (6.21 nm). The corresponding energy dispersive spectroscopy (EDS) elemental maps of the GF separator indicate that the main components of GF fibers are Si and O, with relatively low C content (Figure S3). In contrast, the corresponding EDS elemental maps of the ND-GF separator show a higher C content, indicating that high-density NDs have a more uniform distribution on the surface of the GF separator (Figure 1e). In addition, the wettability of different separators on electrolytes was studied through contact angle tests (Figure S4). The contact angle for the GF separator and NDs-GF separator shows a lower contact angle (close to 0°), indicating that NDs do not affect the wettability of the GF separator material. They both have good wettability, which is beneficial for improving battery performance.^26^

**Figure 2:**
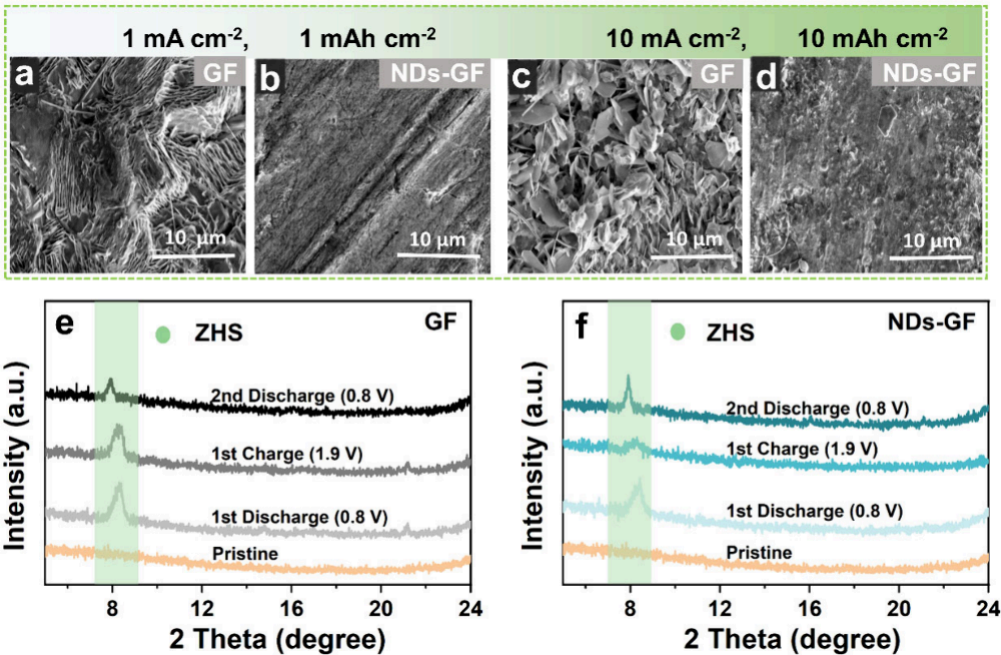
SEM images of the Zn anodes in Zn||Zn symmetric cells under a current density of 1 mA cm^–2^ and 10 mA cm^–2^ respectively. (a, c) The Zn anodes using GF separators, (b, d) the Zn anodes using NDs-GF separators. Different potential states of *Ex Situ* XRD spectra for MnO_2_ cathodes after cycling at 2 A g^–1^ in Zn||MnO_2_ full cells were determined using (e) the GF separator and (f) the NDs-GF separator.

The surface morphology of the Zn anode after cycling to 10th in the symmetric Zn||Zn cell assembled with different separators was observed by SEM image. After 10 cycles under the current density of 1 mA cm^–2^ for a capacity density of 1 mAh cm^–2^ (Figures 2a and 2b), the surface of the Zn anode using GF separators becomes coated with a large amount of Zn dendrites. This manifestation indicates an uneven Zn^2+^ deposition and substantial surface corrosion.^27^ Observed from the corresponding EDS elemental maps (Figure S5a), the surface of the Zn anode related to GF separator has a higher content of O and S elements, further confirming the higher content of byproducts (Zn_4_(OH)_6_SO_4_·*x*H_2_O, ZHS).^28,29^ In contrast, the surface of the Zn anode with the NDs-GF separator presents a more uniform and sleek appearance and a uniform distribution of Zn elements with relatively low O and S content (Figure S5b), indicating a lower ZHS content. At high current density (cycling for 1 cycle at 10 mA cm^–2^ for 10 mAh cm^–2^), the surface of the Zn anode with GF separators is covered with more densely packed sheet-like dendrites (Figure 2c), which is due to the uncontrollable Zn^2+^ transport causing severe side reactions and producing a large number of byproducts. Importantly, the dendrites and byproducts of Zn anode related to NDs-GF separators are significantly less (Figure 2d), and preliminary results indicate that NDs have a positive impact on inhibiting Zn dendrites and byproducts. After 5 cycles under the current density (10 mA cm^–2^, 10 mAh cm^–2^), the NDs-GF separator was analyzed by SEM and the corresponding EDS elemental maps (Figure S6). The results indicate that NDs have good adhesion on the surface of GF separators, which can effectively improve the performance of the GF separator.

Under different potential states, the MnO_2_ cathodes were tested by X-ray diffraction (XRD) spectra (Figures 2e and 2f) to investigate the influence of the separators on the reversibility of Zn||MnO_2_ full cells. The discharge process involves the coinsertion/deinsertion mechanism of H^+^ and Zn^2+^, producing the discharge products such as ZHS, which will disappear during the charging process.^28,30^ Specifically, during the first full discharge (0.8 V) process, the HER caused by water decomposition resulted in a change in pH value, accompanied by the dissolution reaction of MnO_2_ to generate ZHS, as shown in the Ex Situ XRD spectra (approximately 8.2°). For the first charge process (1.9 V), the formed ZHS dissolves, and the peak intensity decreases, indicating the cell has good reversibility. When the second discharge (0.8 V) occurs, ZHS will reappear, and this cycle repeats to achieve energy storage and conversion. The specific reaction process is as follows:^31^

The first discharge process
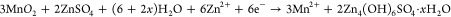
1From the first charge to subsequent cycles
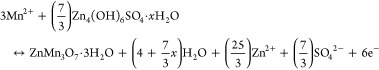
2

By comparison of different potential states of the XRD spectra for MnO_2_ cathodes, the intensity changes of ZHS diffraction peaks in different separators indicate that cells using NDs-GF separators have good reversible cycling stability.

**Figure 3:**
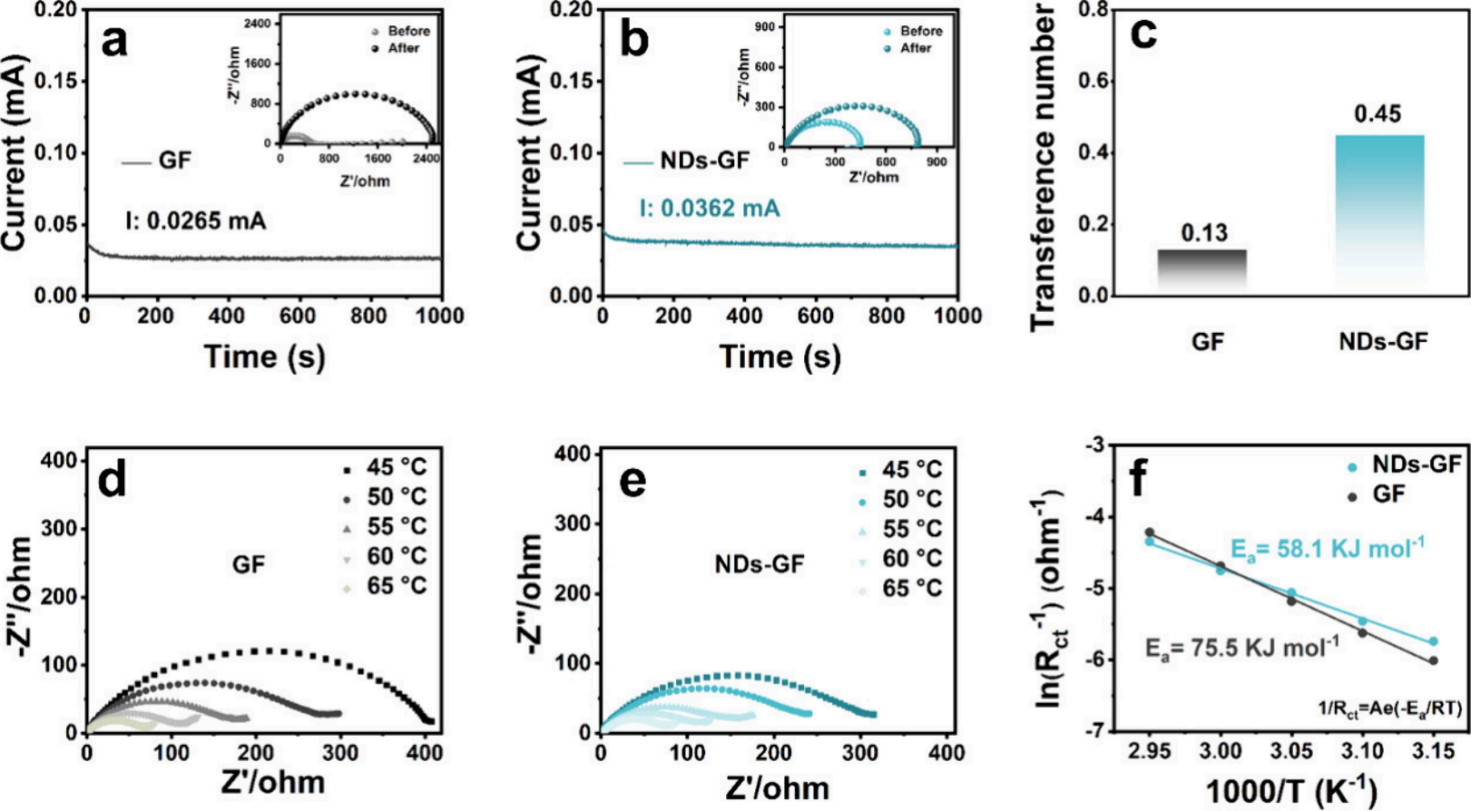
(a, b) Current density–time curves of different Zn||Zn symmetric cells during the polarization tests. The insets in (a) and (b) are EIS curves of the corresponding cells before and after polarization. (c) Corresponding Zn^2+^ transference numbers (*t*_Zn^2+^_). (d, e) EIS curves of different Zn||Zn symmetric cells under different temperatures and (f) the corresponding desolvation activation energy.

As a transport intermediary for Zn^2+^, the separator plays a crucial role in homogenizing the Zn^2+^ concentration and current density. The Zn^2+^ transport kinetics through GF and NDs-GF separators were studied by the Zn^2+^ transference numbers (*t*_Zn^2+^_), which is related to the amperometric *I*–*t* and electrochemical impedance spectroscopy (EIS) (Figures 3a and 3b). The calculation equation is as follows:^15^
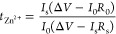
3where *I*_0_ and *R*_0_ are the beginning current and resistance, *I*_s_ and *R*_s_ are the steady-state current and resistance, respectively. The Δ*V* (10 mV) is the voltage polarization applied. Low *t*_Zn^2+^_ can lead to a Zn^2+^ concentration difference at the EEI, resulting in a concentration overpotential and slowing the reaction kinetics. In addition, the Zn^2+^ transport is inevitably accompanied by SO_4_^2–^, and lower *t*_Zn^2+^_ leads to the concentration of SO_4_^2–^ on the surface of Zn, triggering byproducts.^32^ As shown in Figure 3c, the *t*_Zn^2+^_ value of the GF separator is relatively small (0.13), indicating that uneven Zn^2+^ deposition behavior leads to concentration polarization at EEI, intensifying the growth of Zn dendrites and more byproducts. In contrast, the NDs-GF separator has a higher *t*_Zn^2+^_ value (0.45), indicating that the NDs-GF separator enhances Zn^2+^ transport kinetics, thereby alleviating the concentration difference at EEI and effectively suppressing the growth of Zn dendrites and byproducts. The NDs-GF separator increased the Zn^2+^ transport kinetics, possibly due to the abundant −COOH functional groups of NDs’ surface. The strong electrostatic interaction between the unshared electron pair of the −COOH functional group and Zn^2+^ can accelerate the Zn^2+^ transport.^23^ In addition, benefiting from the nanoscale structure, NDs have a large specific surface area, which is conducive to “trapping” enough electrolytes in the pore to achieve rapid Zn^2+^ transport.^33^

The desolvation activation energy (*E*_a_) is also a key parameter reflecting the transport performance of Zn^2+^. Generally speaking, a low *E*_a_ value indicates that hydrated Zn^2+^ only needs to overcome a small energy barrier during the desolvation process, which is beneficial for accelerating Zn^2+^ transport and increasing reaction kinetics.^34,35^ The *E*_a_ of different separators is calculated using the Arrhenius equation (). As shown in Figures 3d and 3e, through the EIS curves of Zn||Zn symmetric cells under different temperatures (45–65 °C), which can obtain the charge transfer resistance (*R*_ct_). As shown in Figure 3f, the calculated *E*_a_ of the NDs-GF separator is 58.1 kJ mol^–1^, which is smaller than the GF separator (75.5 kJ mol^–1^), indicating that NDs facilitate the desolvation of hydrated Zn^2+^ and accelerate the Zn^2+^ transport process. This can also be attributed to the strong electrostatic interaction between −COOH functional groups of NDs’ surface and Zn^2+^, which can regulate the Zn^2+^ solvation structure and effectively facilitate the Zn^2+^ desolvation process at EEI.^23^

**Figure 4:**
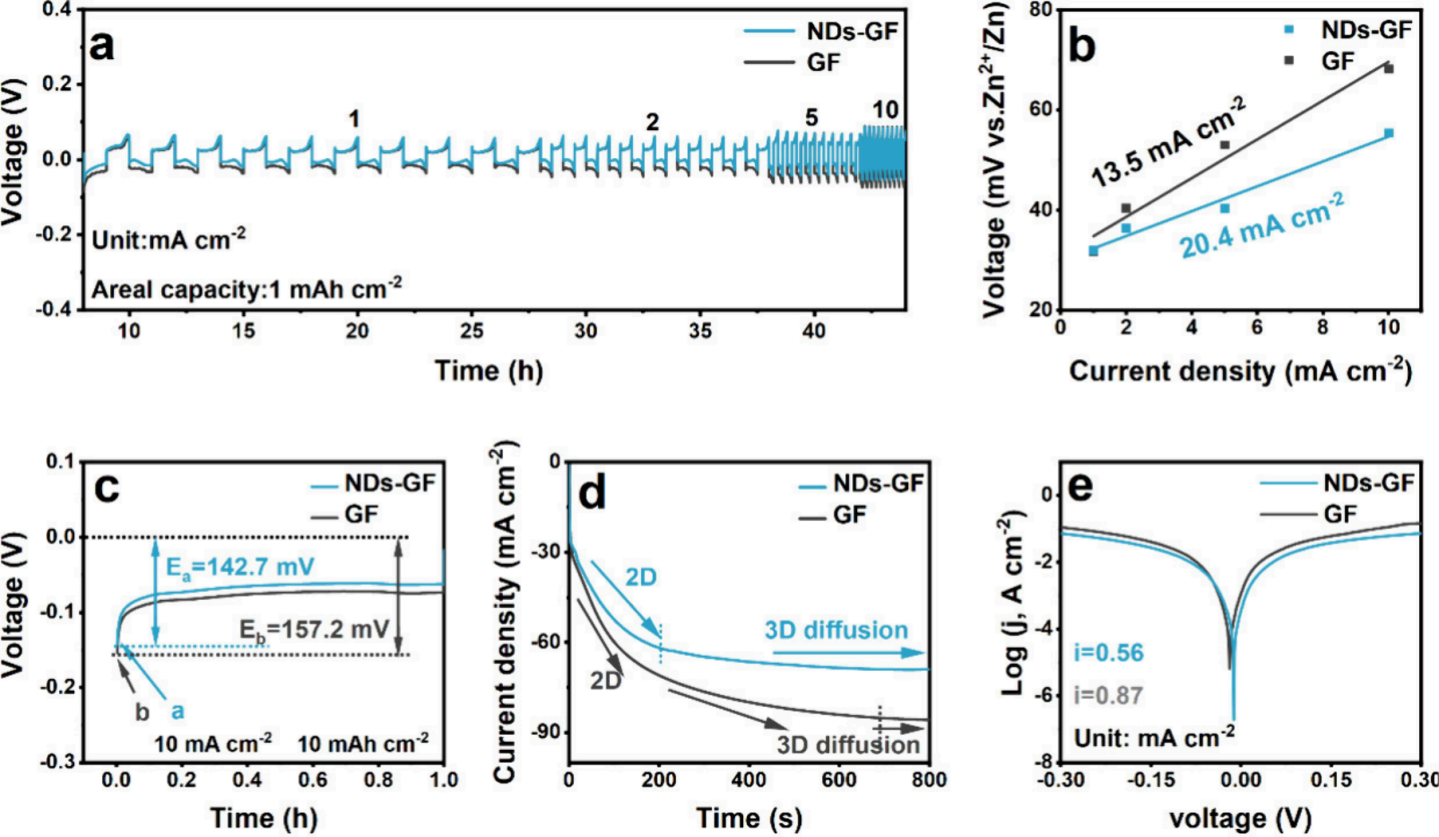
Electrochemical performance of different Zn||Zn symmetric cells (a) Rate performances under different current densities and a fixed capacity of 1 mAh cm^–2^, and (b) the corresponding exchange current density. (c) The first discharge profiles at 10 mA cm^–2^. (d) Chronoamperometry (CA) curves at −150 mV. (e) Tafel plots of NDs-GF and GF separators in Zn||Zn symmetric cells.

Observed from the rate performance of Zn||Zn symmetric cells (Figure 4a), the polarization of the NDs-GF separator is consistently lower than that of the GF separator, reflecting fast kinetics of redox reactions in the cell.^36,37^ The uneven size of GF separator pores causes a nonuniform Zn^2+^ transport rate, leading to significant Zn^2+^ concentration polarization and seriously affecting the electrochemical performance of the cells. The Zn||Zn symmetric cells using NDs-GF separators can maintain stable cycling at each current density, especially at a high current density of 10 mA cm^–2^. This indicates that the NDs-GF separator can not only regulate the Zn^2+^ transport properties, and promote uniform and reversible Zn^2+^ deposition but also enhance the mechanical properties, of the GF separator, maintaining good cycling stability at higher current densities. In order to further study the kinetics of Zn^2+^ deposition, the exchange current density can be calculated by Equation 4.^38^
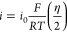
4

The *i*_0_ represents the exchange current density. The *i*, η, *F*, *R*, and *T* are the current density, total overpotential, Faraday constant, gas constant, and absolute temperature, respectively. The Zn||Zn symmetric cells using NDs-GF separators exhibit a higher exchange current density value of 20.4 mA cm^–2^ (Figure 4b). Typically, a higher exchange current density is more favorable for Zn^2+^ deposition, and the results indicate that the presence of NDs promotes uniform Zn^2+^ deposition.^39^ The voltage–time curve of the Zn^2+^ electrodeposition process can provide a deeper understanding of the meaning of deposition kinetics (Figure 4c). With the application of voltage, *E* rapidly increases, and when it reaches *E*_a_ or *E*_b_, this process corresponds to the Zn^2+^ nucleation process, where *E*_a_ and *E*_b_ represent the nucleation overpotential. The Zn^2+^ nucleation overpotential of the NDs-GF separator is 142.7 mV, which is lower than that of the GF separator (157.2 mV). The smaller nucleation overpotential means that a smaller driving force is required for Zn^2+^ to initial nucleation, indicating that the NDs-GF separator improves the Zn^2+^ deposition kinetics.^40^

A chronoamperometry (CA) test was conducted to study the Zn^2+^ deposition behavior (Figure 4d). Under an overpotential of −150 mV, the Zn||Zn symmetric cells using GF separators exhibit an extended and pronounced two-dimensional (2D) Zn^2+^ diffusion process aimed at reducing surface energy,^41^ leading to a continuous rise in current density over 660 s. This progressive increase suggests the existence of surface roughness (Zn dendrites) on the Zn anode surface. The Zn||Zn symmetric cells using NDs-GF separators can maintain a stable current density after the initial deposition period (within 200 s), and the current value is much smaller. This may be explained by the rough surface of the NDs-GF separator, which limits 2D diffusion and quickly forms a stable Zn nucleation. The subsequent stable current density indicates that NDs regulate the uniform Zn^2+^ deposition, providing more active nucleation sites for EEI rather than localized Zn dendrites.^42^ Additionally, from the Tafel plot (Figure 4e), the Zn anode using NDs-GF separators exhibits a smaller corrosion current density (0.56 mA cm^–2^), indicating that NDs-GF separators enhance the corrosion resistance of the Zn anode.^43,44^

**Figure 5:**
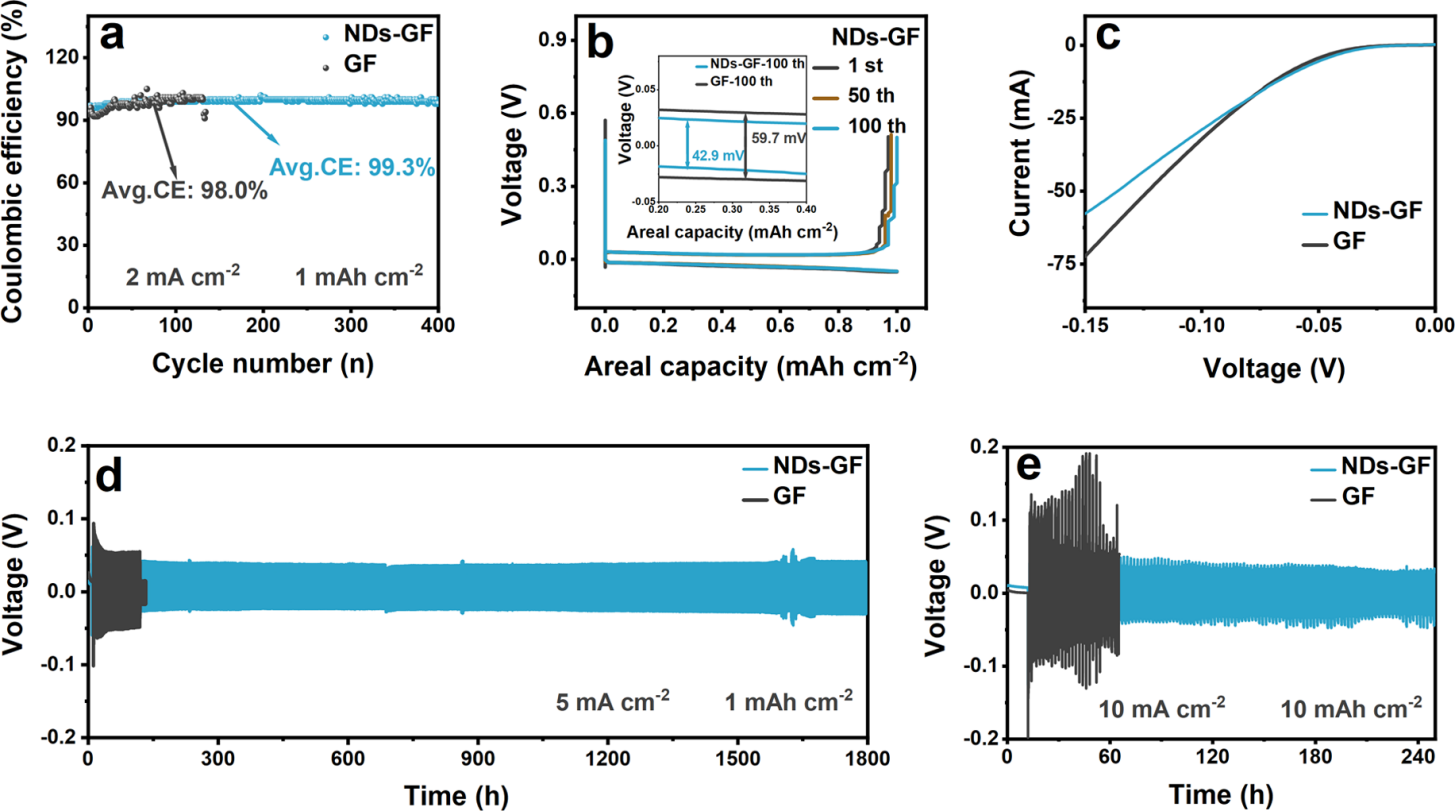
Performance of NDs-GF and GF separators in Zn||Cu asymmetric cells (a) the Coulombic efficiency (CE), (b) charge–discharge cycle curves, and (c) hydrogen evolution reaction (HER) performance of different Zn||Ti asymmetric cells. The cycling performance of NDs-GF and GF separators in Zn||Zn symmetric cells under (d) 5 mA cm^–2^ and 1 mAh cm^–2^, and (e) 10 mA cm^–2^ and 10 mAh cm^–2^.

For the electrochemical reversibility of Zn||Cu asymmetric cells under a current density of 2 mA cm^–2^ for a capacity density of 1 mAh cm^–2^ (Figure 5a), the cell using the NDs-GF separator runs for over 400 h with a high average Coulombic efficiency (CE) of 99.3%. In contrast, the Zn||Cu asymmetric cells using GF separators display pronounced CE fluctuations throughout the cycles, suggesting inadequate reversibility attributed to side reactions and dendrite formation.^45^ Additionally, the NDs-GF separator has lower overpotential (42.9 mV) and overlapping voltage curves for the selected cycles, which indicates faster ion diffusion kinetics and the reversible Zn stripping/plating process at EEI (Figures 5b and Figure S7).^46,47^ To reveal the inhibition of NDs-GF separators on the HER, linear sweep voltammetry (LSV) testing was conducted on different Zn||Ti asymmetric cells, as shown in Figure 5c. The hydrogen evolution overpotential of the Zn||Ti asymmetric cells using NDs-GF separators show a significant negative shift, indicating that HER in the electrolyte is particularly difficult to occur.^48^ That is due to the abundant hydroxyl (−OH) functional groups on the surface of NDs, which can form hydrogen bonds with H^+^ to reduce the H^+^ concentration at EEI to some extent, thereby inhibiting HER.^22^

As anticipated, the NDs modified GF separators contribute to the long-term stable cycling of Zn||Zn symmetric cells. The cycling curves, validated in the Zn||Zn symmetric cells using different separators, are depicted in Figures 5d and 5e. The Zn||Zn symmetric cells using NDs-GF separators exhibit a remarkable cycle life of nearly 1800 h at a current density of 5 mA cm^–2^, more than 16 times that of the GF separator (110 h). Compared with other reported separators, this study has significance in extending the cycle life of cells (Table S1).^15,49−56^ Especially under the high current density of 10 mA cm^–2^ and 10 mAh cm^–2^, the Zn||Zn symmetric cells using NDs-GF separators maintain stable reversible Zn^2+^ plating/stripping for approximately 240 h.^57,58^ That is due to the excellent mechanical properties of NDs, which improve the mechanical performance of GF separators and enable cells to maintain good cycling stability under high current density. In contrast, the GF separator exhibits significant voltage fluctuations, indicating the unstable Zn^2+^ deposition kinetics at high current densities. This is consistent with the stress performance test results in Figure S8. To study the optimal content of NDs, we optimized the content of NDs in GF separators and assembled the corresponding Zn||Zn symmetric cells, as shown in Figure S9 and Table S2. At the initial stage of the cycle, the low-content NDs exhibit significant potential fluctuations, while the high-content NDs exhibit higher potentials. This indicates that low-content NDs did not play a positive regulatory role in the transport properties of Zn^2+^, and their reaction kinetics remain unstable. However, high-content NDs increase Zn^2+^ concentration polarization, leading to higher polarization overpotentials and slowing kinetic performance.^37^ The GF separator modified with medium-content NDs (NDs-GF) exhibits a small and stable potential, which is profitable for the rapid and stable Zn^2+^ plating/stripping process.

**Figure 6:**
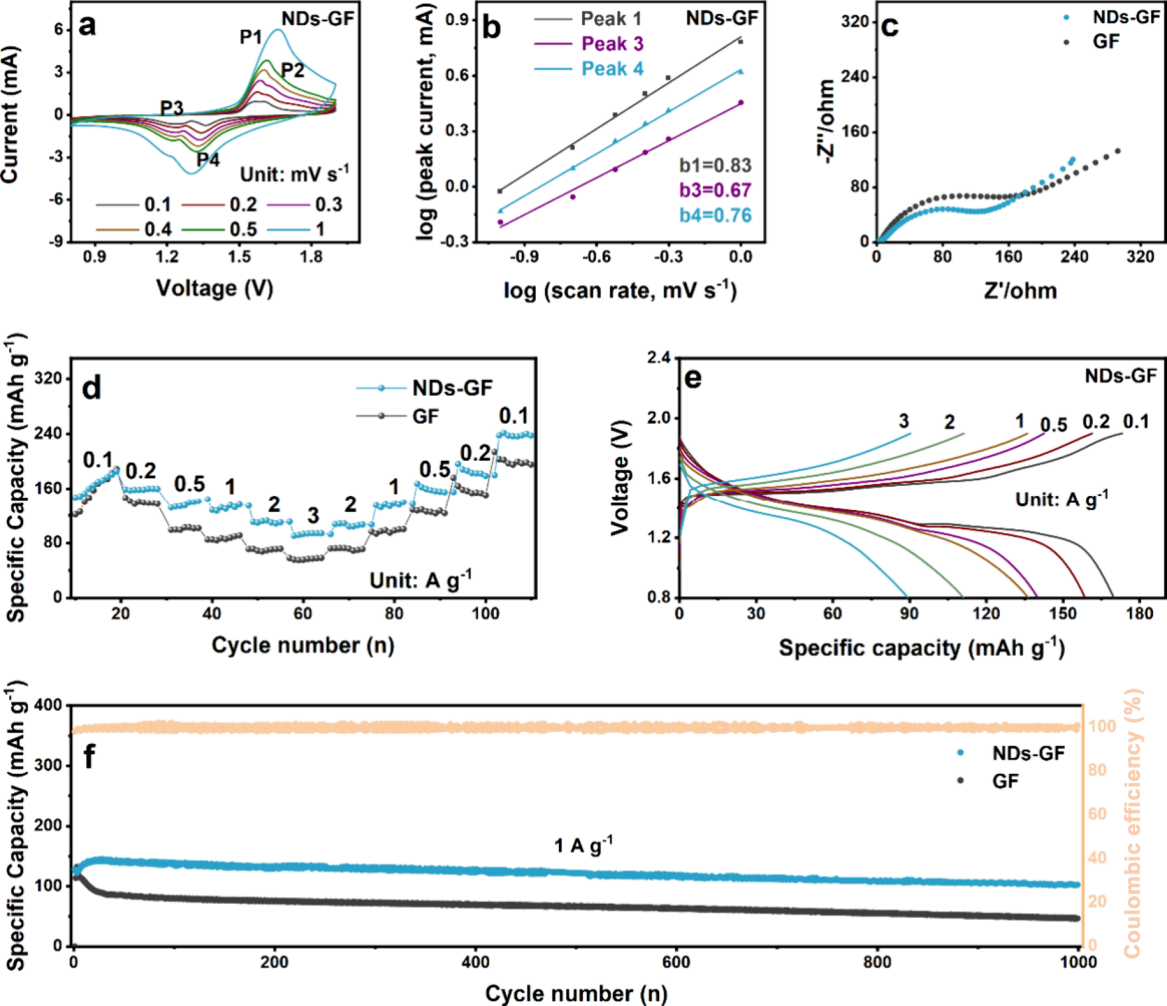
Electrochemical performance of NDs-GF and GF separators in Zn||MnO_2_ full cells. (a) CV curves of NDs-GF separator under different scanning rates. (b) log(*i*) vs log(*v*) plots of the corresponding peaks from CV curves. (c) EIS curves. (d) Rate performance under different current densities. (e) Charge–discharge cycle curves of NDs-GF separator. (f) Cyclic stability and efficiency under the current density of 1 A g^–1^.

Full cells of Zn||MnO_2_ assembled with the NDs-GF separator were tested to evaluate their improved electrochemical properties. As shown in Figure 6a, the CV curves were conducted at various scan rates. The relationship between the peak current and the scanning rate is as follows:^59,60^
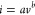
5
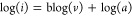
6Where *i* and *v* are the current and scanning rate, *a* and *b* are the corresponding adjustable parameters. The adjustable parameter *b* for peaks 1, 3, and 4 can be obtained by fitting log(*i*) versus log(*v*). The calculated *b* values for peak 1, peak 3, and peak 4 of the Zn||MnO_2_ full cells using NDs-GF separators are theoretically 0.83, 0.67, and 0.76, respectively (Figure 6b), (the peak intensity of 2 cannot be tested under the high scanning rates). The results indicate that based on the large ratio of peak 1, and peak 4, the kinetic reaction is mainly based on the pseudocapacitance process.^61^ In contrast, the Zn||MnO_2_ full cell using the GF separator also exhibited the same CV curve and peak value (Figure S10), but the corresponding b value was smaller, indicating a lower pseudocapacitance. The Zn||MnO_2_ full cell using NDs-GF separator has higher pseudocapacitance, indicating that NDs-GF has good rate performance.^62^ The EIS measurement was utilized to investigate the EEI transport properties of different Zn||MnO_2_ full cells during continuous Zn^2+^ plating/stripping, as shown in Figure 6c. Before cycling, in the high-frequency region, both plots exhibit a depression semicircle associated with the interfacial charge transfer resistance (*R*_ct_). According to the equivalent circuit model (Figure S11), the fitted *R*_ct_ value for the NDs-GF separator is 101.3 ohm, whereas for the GF, the fitted *R*_ct_ value is 117.7 ohm. This can be attributed to the lower interfacial resistance of NDs, which can regulate the Zn^2+^ transfer rate without hindering charge transfer.^16^

As shown in Figure 6d, the Zn||MnO_2_ full cells using NDs-GF separators exhibit outstanding rate performance, attributed to fast reaction kinetics and excellent Zn^2+^ plating/stripping process.^63^ Under the current density from 0.1 A g^–1^ to 3 A g^–1^, the NDs-GF separator demonstrate excellent reversible specific capacities of 185.5, 160.1, 141.4, 135.2, 112.8, and 94.0 mAh g^–1^, respectively. In contrast, the Zn||MnO_2_ full cells using GF separators exhibit relatively lower reversible specific capacities (189.0, 139.3, 101.2, 87.7, 69.3, and 57.3 mAh g^–1^ respectively). When the current density returns to 0.1 A g^–1^, the Zn^2+^ storage capacity of the NDs-GF separator is 240 mAh g^–1^, also surpassing the other (197.1 mAh g^–1^), maintaining a good rate performance. The galvanostatic charge–discharge cycle profiles in Figures 6e and S12 show both cells displaying two different incline charge–discharge curves, which are relevant to the coinsertion/deinsertion mechanism of H^+^ and Zn^2+^, consistent with the CV curves and previous research findings.^64^ Furthermore, as shown in Figure 6f, the long cycling stability of NDs-GF and GF separators in Zn||MnO_2_ full cells was tested at 1 A g^–1^. The Zn||MnO_2_ full cells using NDs-GF separators exhibit high retention and an enduring reversible capacity of 102.4 mA h g^–1^ over more than 1000 cycles. This achievement is attributed to the introduction of NDs-GF separators and the dendrite-free Zn anode. All the aforementioned results highlight the substantial potential of NDs-GF separators in inhibiting Zn dendrites and ensuring the consistent cycling stability of AZIBs.

## 4 Conclusions

In summary, we introduce NDs with the properties of chemical inertness, superhardness, ultrahigh thermal conductivity, and abundant oxygen-containing functional groups to modify GF separators for achieving high-performance AZIBs. The highly dense superhard NDs with chemical inertness filled in GF separators are beneficial for realizing uniform Zn^2+^ transport/deposition and inhibiting dendrite growth. The functional NDs’ surface can accelerate Zn^2+^ transport, assist in Zn^2+^ desolvation, inhibit HER, and reduce byproducts. This study offers a feasible and efficient structural design for optimizing the performance of AZIBs and provides an approach for achieving high-performance commercial AZIBs in the future.

## Data Availability

The data that support the findings of this study are available from the corresponding author upon reasonable request.

## Supporting Information Available

Supporting Figures S1–S12, including TEM and HRTEM images, SEM and EDS images, pore size distribution, water contact angles, tensile performance, and further electrochemical performance data (Cycling performance, CV and EIS curves). Supporting Table S1 and Table S2, including the performance comparison data of NDs-GF with other reported ZIBs separators and the NDs content parameters in different NDs-GF separators (PDF)
