# Supplementary material for: Modulating Ion Behavior by Functional Nanodiamond Modified Separator for High-Rate Durable Aqueous Zinc-Ion Battery
Source: ACS Appl Mater Interfaces. 2024 Dec 9;16(50):69388–97. doi: 10.1021/acsami.4c15737 (PMC11660037; doi:10.1021/acsami.4c15737)
Supplement: Supplementary file 1 — am4c15737_si_001.pdf [file am4c15737_si_001.pdf]

Supporting Information

Modulating Ion Behavior by Functional Nanodiamond Modified Separator for High-Rate Durable Aqueous Zinc-ion Battery

Qiuxia Zhang,[1] Linfeng Wan,[1] Xuan Gao*,[2,3] Shaoheng Cheng,[1] Nan Gao,[1] Claire J. Carmalt,[2] Yuhang Dai*,[3] Guanjie He*,[2] Hongdong Li*[1]

[1] *State Key Laboratory of Superhard Materials, College of Physics, Jilin University, Changchun 130012, PR China*

[2] *Christopher Ingold Laboratory, Department of Chemistry, University College London, London, WC1H 0AJ, UK*

[3] *Thom Building, Department of Engineering Science, University of Oxford, Oxford, OX1 3PJ, UK*

**Corresponding author:*

xuan.gao@eng.ox.ac.uk, whutdyh@gmail.com, g.he@ucl.ac.uk, hdli@jlu.edu.c

This PDF file is 8 pages and mainly includes characterization figures, electrochemical performance data, a performance comparison between NDs-GF separators and reported ZIBs separators, and NDs content parameters in NDs-GF separators.

Number of Figure: 12

Number of Table: 2

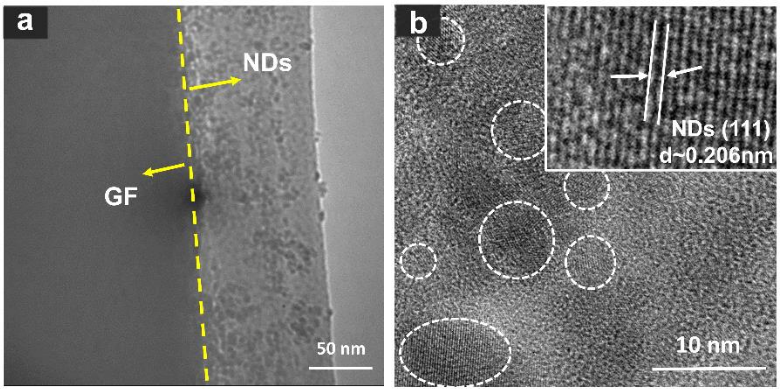

Figure S1. (a) TEM and (b) HRTEM images of the NDs-GF separator.

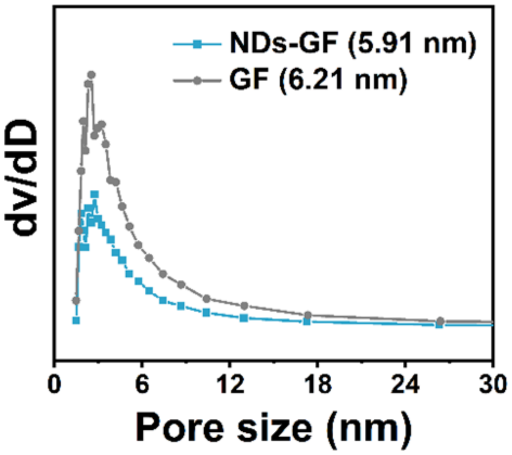

Figure S2. The pore size distribution of the NDs-GF and GF separators.

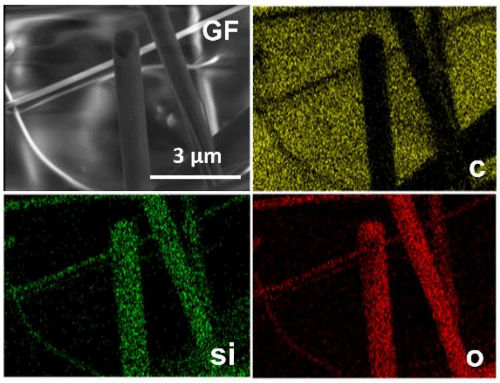

Figure S3. SEM image and the corresponding EDS of the GF separator.

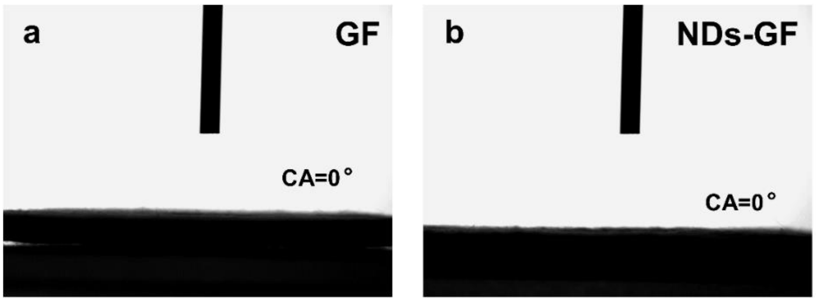

Figure S4. The water contact angles of (a) GF separator and (b) NDs-GF separator.

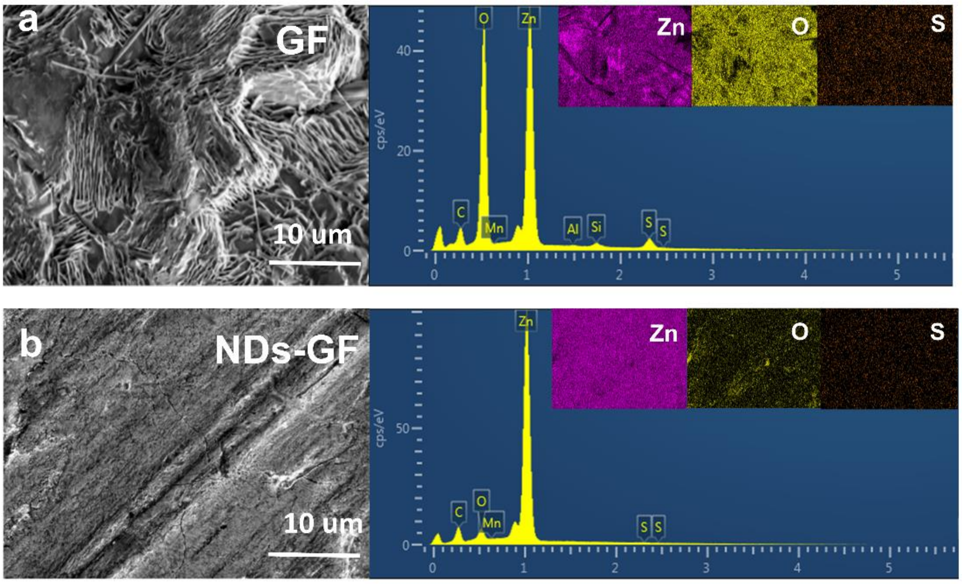

Figure S5. SEM images and the corresponding EDS of Zn anodes after 10 cycles under the current density of 1 mA cm^{-2} and 1 mA cm^{-2} (a) GF separator and (b) NDs-GF separator.

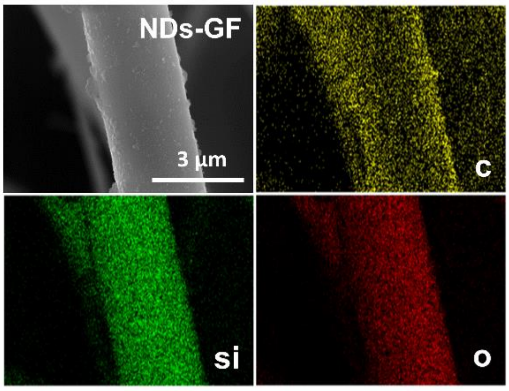

Figure S6. SEM image and the corresponding EDS of the NDs-GF separator in Zn||Zn symmetric cells after 5 cycles.

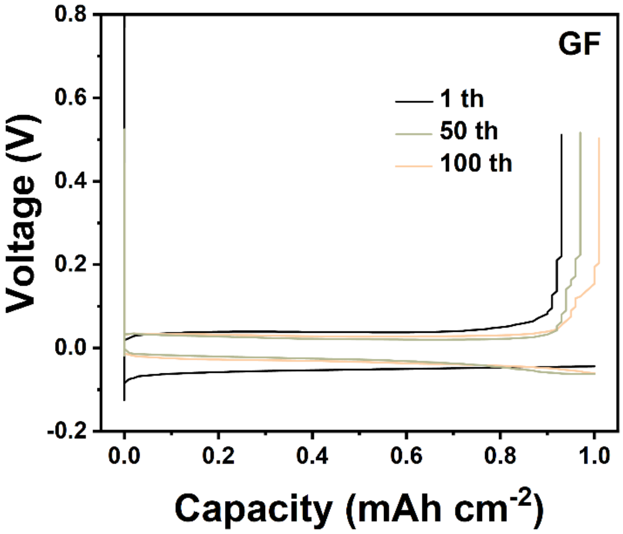

Figure S7. Charge-discharge profiles of the GF separator in Zn||Cu asymmetric cell.

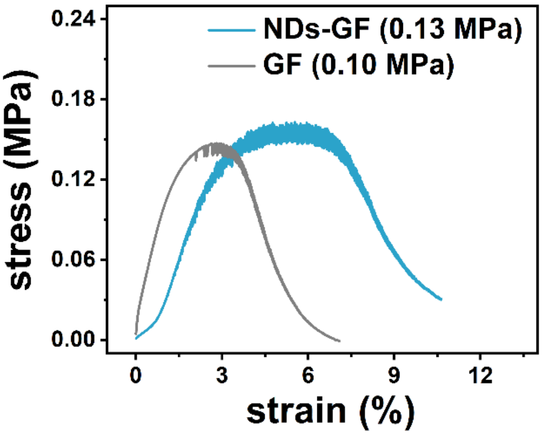

Figure S8. Tensile curves of the NDs-GF and GF separators.

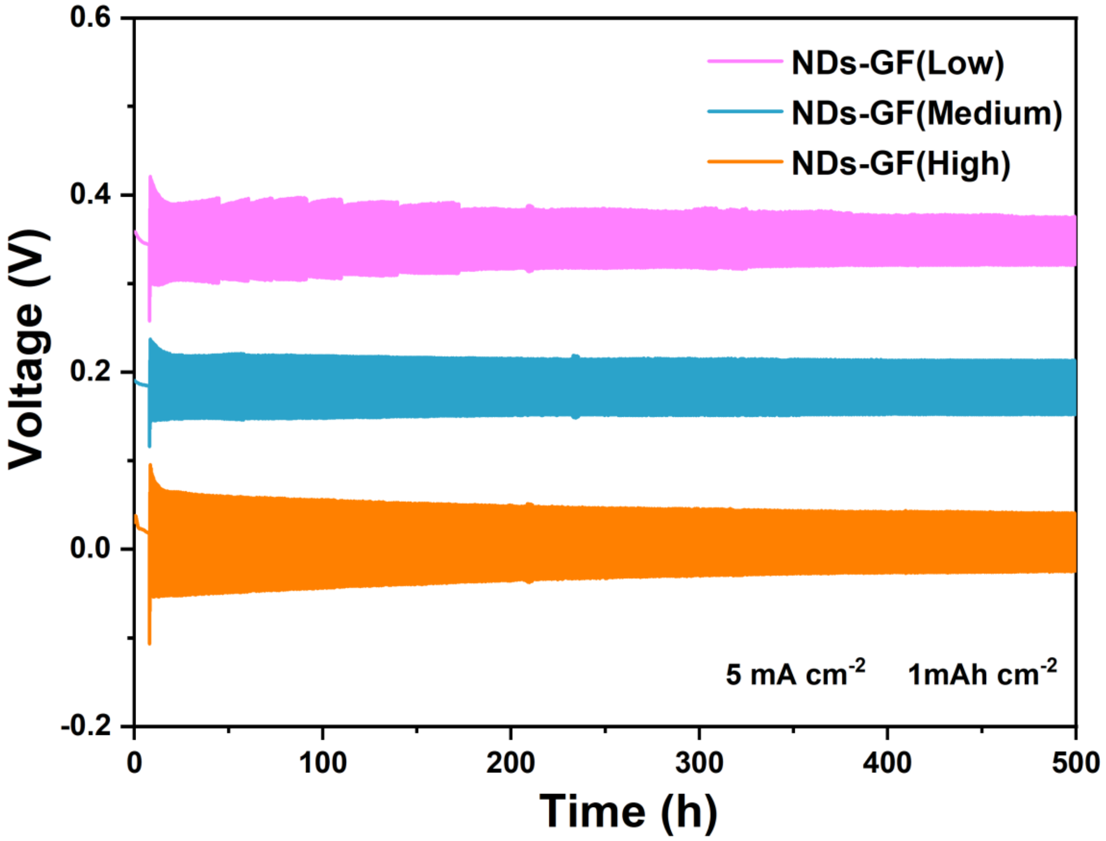

Figure S9. Cycling performance of Zn||Zn symmetric cells using different content of NDs in GF separators under 5 mA cm^{-2} for 1 mAh cm^{-2}.

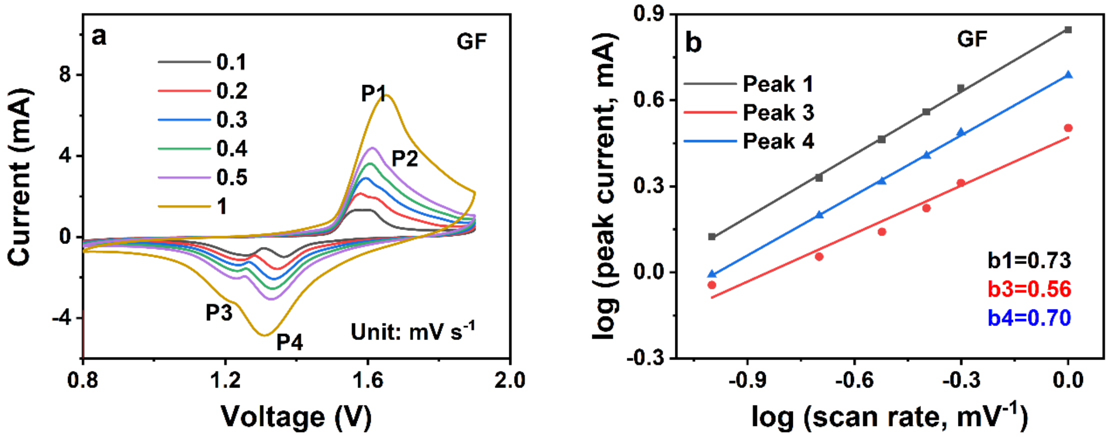

Figure S10. (a) CV curves of GF separator in Zn||MnO_2 full cell under the scan rate from 0.1 to 1.0 mv s^{-1}. (b) Log (i) vs log (v) plots of the corresponding three peaks from CV curves.

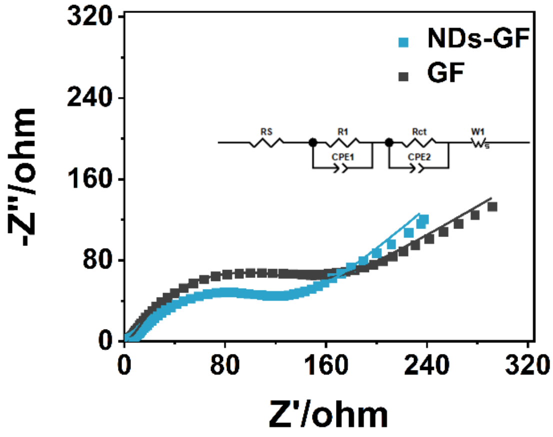

Figure S11. The equivalent circuit model of EIS curves for Zn||MnO_2 full cells using NDs-GF and GF separators.

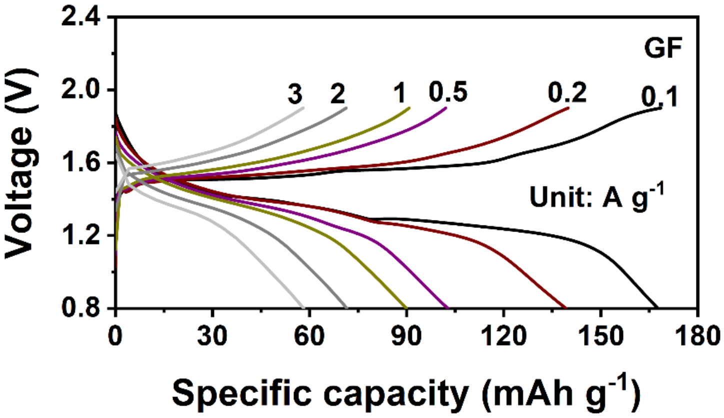

Figure S12. Charge-discharge profiles of rate performance using GF separator.

Table S1. Performance comparison of NDs-GF with other ZIBs separators was recently reported.

Materials	Current density (mA cm^{-2})	Capacity density (mAh cm^{-2})	Cycle life (h)	Ref
NDs-GF	5	1	1800	This work
MXene-GF	5	1	1200	[1]
UiO-66-GF-2.2	2	1	1650	[2]
CCM	5	1	600	[3]
PANI-GF	2	1	560	[4]
A-TiO2@GF	1	1	1500	[5]

TTA-DHTPA-COF@GF@Gr	5	1	600	6
LA@GF	5	1	250	7
ZIF-8-GF	2	1	840	8
SR-GF	4	1	400	9

Table S2. The mass loading of NDs in the separator.

Samples	1 (mg cm^{-2})	2 (mg cm^{-2})	3 (mg cm^{-2})	Average value (mg cm^{-2})
NDs-GF (Low)	0.0498	0.1990	0.0995	0.116
NDs-GF (Medium)	0.2496	0.2985	0.2985	0.282
NDs-GF (High)	0.4478	0.5473	0.5473	0.514

References

(1) Su, Y.; Liu, B.; Zhang, Q.; Peng, J.; Wei, C.; Li, S.; Li, W.; Xue, Z.; Yang, X.; Sun, J. Printing-Scalable $Ti_3C_2T_x$ MXene-Decorated Janus Separator with Expedited Zn^{2+} Flux toward Stabilized Zn Anodes. *Adv. Funct. Mater.* **2022,** *32* (32), No. 2204306.

(2) Song, Y.; Ruan, P.; Mao, C.; Chang, Y.; Wang, L.; Dai, L.; Zhou, P.; Lu, B.; Zhou, J.; He, Z. Metal–Organic Frameworks Functionalized Separators for Robust Aqueous Zinc-Ion Batteries. *Nano-Micro Lett.* **2022,** *14* (1), No. 218.

(3) Zhang, Y.; Liu, Z.; Li, X.; Fan, L.; Shuai, Y.; Zhang, N. Loosening Zinc Ions from Separator Boosts Stable Zn Plating/Striping Behavior for Aqueous Zinc Ion Batteries. *Adv. Energy Mater.* **2023,** *13* (42), No. 2302126.

(4) Zhao, N.; Zhang, Y.; Zhang, Z.; Han, C.; Liang, Y.; Li, J.; Wang, X.; Dai, L.; Wang, L.; He, Z. Polyaniline Functionalized Separator as Synergistic Medium for Aqueous Zinc-Ion Batteries. *J. Colloid Interface Sci.* **2023,** *642*, 421-429.

(5) Lv, S.; Su, M.; Li, Z.; Mao, Y.; Yin, J.; cao, D.; Wang, G.; Yi, J.; Ning, F.; Zhu, K. Hydrophilic-Zincophobic Separator Enabling by Crystal Structure Regulation toward Stabilized Zn Metal Anode. *Adv. Funct. Mater.* **2024,** *34* (27), No. 2315910.

(6) Huang, T.; Wang, S.; Wu, J.; Hu, H.; Wang, J.; Zhang, X.; Gao, Y. Triazine and Hydroxyl Covalent Organic Framework Modified Separator for Zn-Ion Fast and Selective Transport and Dendrite-Free Deposition in Zinc–Iodine Battery. *J. Power Sources* **2024,** *608*, No. 234658.

(7) Li, R.; Xiang, T.; Wang, P.; Gong, Z.; Wu, Y.; Zhang, Y.; Shi, F.; Zhou, M. Regulation of the Anode Electrodeposition Behavior of Aqueous ZincIon Batteries by an l-Alanine-Modified Glass Fiber Separator. *Ind. Eng. Chem. Res.* **2024,** *63* (37), 16164-16173.

(8) Zhang, W.; Zhu, X.; Kang, L.; Peng, Z.; Zhu, J.; Pan, L.; Dai, L.; Liu, S.; Wang, L.; Liu, Y.; He, Z. Stabilizing Zinc Anode using Zeolite Imidazole Framework Functionalized Separator for Durable Aqueous Zinc-Ion Batteries. *J. Energy Chem.* **2024,** *90*, 23-31.

(9) Sun, Z.; Zhang, J.; Jiao, X.; Li, Z. A Low-Cost Biomass-Derived Carbon for High-Performance Aqueous Zinc Ion Battery Diaphragms. *J. Energy Storage* **2024,** *100*, No. 113780.
